# Functional Reconstitution of *Staphylococcus aureu*s Truncated AgrC Histidine Kinase in a Model Membrane System

**DOI:** 10.1371/journal.pone.0080400

**Published:** 2013-11-26

**Authors:** Lina Wang, Chunshan Quan, Baoquan Liu, Jianfeng Wang, Wen Xiong, Pengchao Zhao, Shengdi Fan

**Affiliations:** 1 Dalian Institute of Chemical Physics, Chinese Academy of Sciences, Dalian, China; 2 Department of Life Science, Dalian Nationalities University, Dalian, China; 3 The State Ethnic Affairs Commission-Ministry of Education, Dalian, China; The Scripps Research Institute and Sorrento Therapeutics, Inc., United States of America

## Abstract

The integral membrane protein AgrC is a histidine kinase whose sensor domains interact with an autoinducing peptide, resulting in a series of downstream responses. In this study, truncated AgrC_TM5-6C_ and AgrC_TM5-6C_-GFP with GFP as a reporter gene were produced using a bacterial system. Purified AgrC_TM5-6C_ and AgrC_TM5-6C_-GFP were reconstituted into liposomes by a detergent-mediated method. To achieve high-yield protein incorporation, we investigated the effect of different detergents on protein reconstitution efficiency. The highest incorporation was found with N,N-dimethyldode-cylamine N-oxide during complete liposome solubilization, which resulted in a yield of 85±5%. The COOH-terminus of the protein AgrC_TM5-6C_ was almost exclusively oriented towards the inside of the vesicles. AgrC_TM5-6C_ in proteoliposomes exhibited approximately a 6-fold increase in constitutive activity compared with AgrC_TM5-6C_ in detergent micelles. The reconstitution of AgrC_TM5-6C_ or AgrC_TM5-6C_-GFP was characterized using dynamic light scattering, fluorescence microscopy, and transmission electron microscopy. Based on the results, the optimal conditions for protein incorporation were defined. These findings contribute to the study of membrane protein structure and function *in vitro* using a reconstitution system.

## Introduction

Two-component signal transduction (TCST) is a universal and important microbial modality for sensing and responding to diverse environmental changes. Although TCST systems are also found in plants, fungi, and other protists, they are absent in animals. Therefore, these systems are potential targets for the development of novel antibiotics [1], [2]. The classical TCST system consists of a transmembrane histidine protein kinase (HPK) receptor and a cytoplasmic response regulator (RR). Extracellular domain of the HPK senses an external signal and transmits it to the RR by conserved phosphotransfer events, resulting in an intracellular response [3], [4]. HPK receptors are reported to function as dimers [5], [6], however, the molecular mechanism for signal transduction across cell membranes remains unknown.

*Staphylococcus aureus*, a human pathogen, is a major problem of hospitals and a cause of infections in otherwise healthy individuals [7], [8]. The spectrum of diseases caused by *S. aureus* ranges from superficial skin infections to life-threatening disease [9]. Even with antibiotics, the morbidity and mortality associated with *staphylococcal* infections is high. Some strains are resistant to methicillin and oxacillin and the species in general has high virulence and transmissibility [10]. Consequently, novel antibacterial targets and new agents that attenuate virulence and disrupt the capacity of pathogenic bacteria to cause infection are urgently needed [11]. Virulence in *S. aureus* is largely regulated by the accessory gene regulator (*agr*) quorum-sensing system. Thus, *agr* is a focus as a new antibiotic target. Gordon et al. described new antibacterial targets and agents directed towards the (1) *agr* quorum-sensing system, (2) the transcriptional activator AgrA−DNA, (3) RNAIII, and (4) the SarA family of transcriptional regulators [12].

The *agr* locus of *S. aureus* is composed of two divergent transcription units named RNAII and RNAIII, controlled by the respective promoters P2 and P3 [13]. The P2 operon consists of four genes, *agr*BDCA, which are required for activation of transcription from P2 and P3 promoters. The P3 transcript, RNAIII, has 517 nucleotides and is the intracellular effector of the *agr* response [14], [15]. AgrA and AgrC constitute a two-component system with AgrC as the sensor histidine kinase and AgrA as the response regulator. AgrC, a 45 kDa integral membrane protein, is a member of the class 10 HPK family [16]. AgrA is the only member of the LytTR class of response regulator for which a structure has been determined [17]. Although many studies have illuminated ligand specificity determinants in AgrC [18]–[20], questions remain unresolved about how signal molecules affect AgrC kinase activity and how conformation associated with the regulation of AgrC kinase activity changes after autophosphorylation. In particular, little is known about the specific regions of AgrC that form dimers or oligomers during signal transmission. Similar to AgrC, many two-component sensor kinases have multiple membrane-spanning domains and are located in complex biological membranes, making study of their *in situ* structure and function difficult. Incorporation of membrane proteins purified from the native cell membranes into an artificial lipid bilayer is an excellent *in vitro* tool for elucidating of membrane protein structure and function. An artificial membrane system was recently used to analyze the activity of all membrane sensor kinases from *Entercoccus faecalis* [21] and to functionally reconstitute the thermosensor DesK of *Bacillus subtilis* [22], PhoQ of *Salmonella typhimurium* [23], and KdpD, EnvZ and DcuS of *E. coli* [24]–[26]. Reconstitution technology has the advantages of simplifying the complexity of biological systems and allowing control of *in vitro* system conditions and components, which potentially permits the precise study of receptor oligomerization and molecular mechanisms of ligand-receptor interactions.

To date, membrane proteins have been inserted into liposomes using strategies that involve mechanical means, freeze-thawing, organic solvents, or detergents. Successful incorporation of proteins into liposomes has largely used detergent-mediated methods [27], [28]. Previous studies have not found a single detergent that works equally well to reconstitute all membrane proteins [29]–[31]. Therefore, screening a suitable detergent is essential for a successful reconstitution strategy.

The goal of this work was to develop an effective method for constructing proteoliposomes. Our methodological approach could be useful for functional studies of membrane proteins in model systems. Truncated AgrC (residues 134–430), which has two transmembrane domains, an extracellular loop, and a cytoplasm domain, was used as a model protein because the truncated AgrC retains the constitutive kinase functions. The initial establishment of our reconstitution methods required a large amount of membrane protein and yields of expressed truncated AgrC (AgrC_TM5-6C_) are higher than yields of full-length AgrC. Incorporation of the recombinant AgrC_TM5-6C_-GFP into lipid membranes was used to evaluate protein reconstitution efficiency by centrifugation followed by fluorescence intensity measurement. Transitional changes induced by the interaction of detergents with phospholipids were studied by means of light-scattering and transmission electron microscopy (TEM). Turbidity data was used to define the steps of the solubilization process. Structures of proteoliposomes were directly visualized using fluorescence microscopy (FM) and TEM. In addition, we characterized the kinase activities of the purified protein reconstituted into phospholipids. These results provided direct evidence that AgrC_TM5-6C_ was reconstituted into model membranes. The construction of an artificial signal transduction model might help further our understanding of signal transmission mechanisms of receptor proteins and be useful for screening signal transfer inhibitors. Thus, the model described here could be important for both determining the role of AgrC_TM5-6C_ plays in signaling and for developing novel antibacterial targets and agents.

## Materials and Methods

### Chemicals and Reagents

Reagents n-dodecylmaltoside (DDM), N,N-dimethyldode-cylamine N-oxide (LDAO) 3-[(3-cholamidopro-pyl)-dimethylammonio]-propanesulfonate (CHAPS), sodium cholate (SC), and n-dodecylphosphocholine (DPC) were from Sigma. Dioleoyl-phosphatidyl-choline (DOPC), 1,2-dipalmitoyl-sn-glycero-3-phosphocholine (DPPC), L-α-phosphatidic acid (egg PA), and cholesterol were from Avanti Polar Lipids. Bio-beads SM2 (Bio-Rad) were washed in methanol and rinsed with double-distilled water before use. Fluorescent reagents 4-acetamido-4’-maleidylstibene-2,2’-disulphonic acid (AmdiS), N-ethylmaleimide (NEM), and 5-iodoacetamidefluorescein (5-IAF) were from Invitrogen. Kinase-Glo Luminescent Kinase Assay Kit was from Promega. Double-distilled water was autoclaved before use. All other chemicals were of the highest purity.

### Membrane protein overexpression and purification

**Figure 1:**
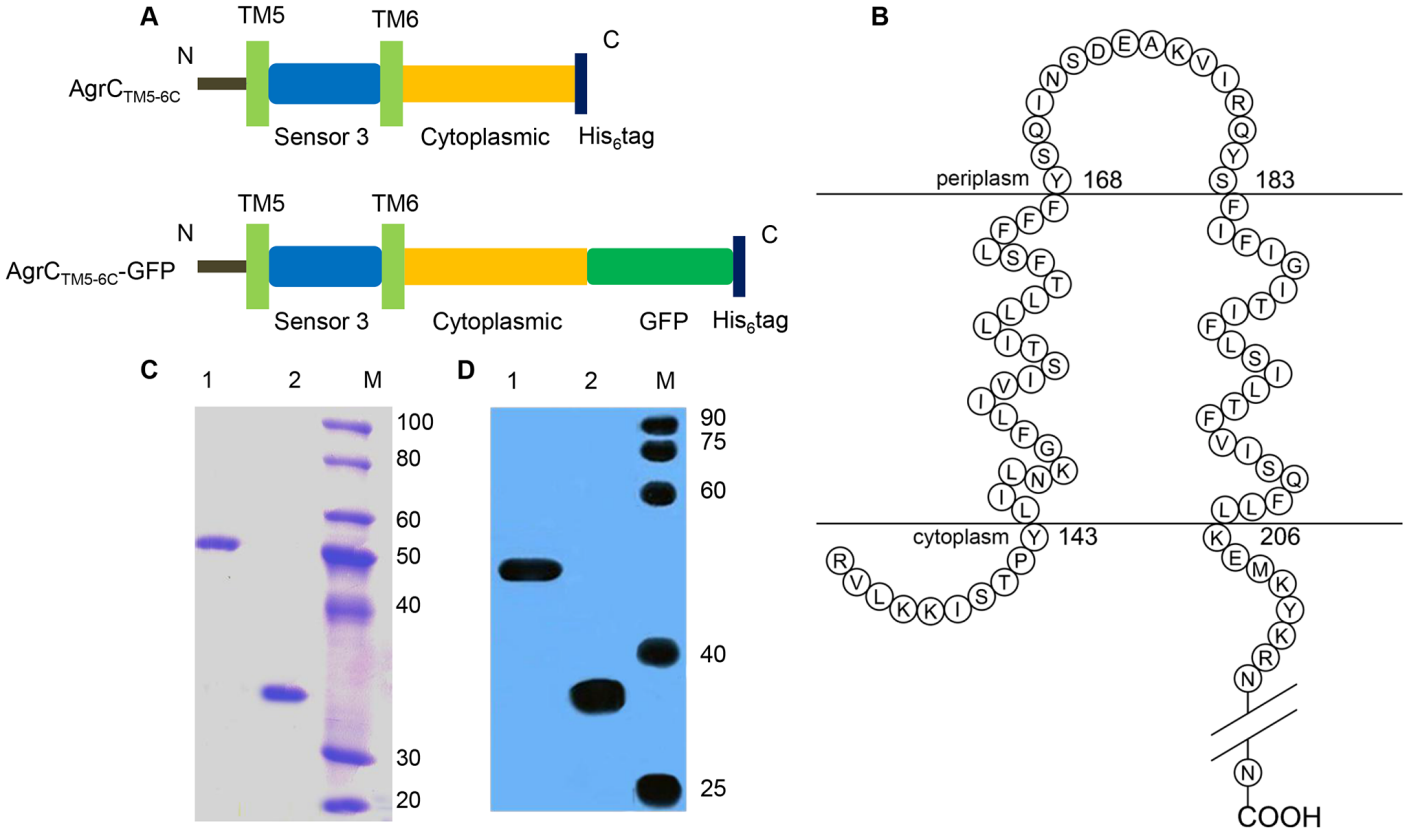
Schematic representation of target protein overexpression and purification. (A) Untagged AgrC_TM5-6C_ and fluorescence-tagged AgrC_TM5-6C_. Genes for GFP were introduced into AgrC_TM5-6C_ constructs, to yield AgrC_TM5-6C_-GFP. (B) Membrane topology model of AgrC_TM5-6C._ (C) SDS-PAGE of AgrC_TM5-6C_ and AgrC_TM5-6C_-GFP purification by size exclusion chromatography. 1, AgrC_TM5-6C_-GFP; 2, AgrC_TM5-6C_; M, size marker. (D) Western blotting analysis of AgrC_TM5-6C_ and AgrC_TM5-6C_-GFP purification.

*Escherichia coli* C43 (DE3) cells harboring the indicated pET-28a-AgrC_TM5-6C_ or pET-28-AgrC_TM5-6C_-GFP vector (Fig. 1A) were routinely grown at 37°C. At optical density 600 nm (OD_600_) 0.25–0.35, isopropyl-β-d-thiogalactoside (IPTG) was added at a final concentration of 0.1 mM to induce protein production from the plasmids. Following 24 h at 20°C, cells were harvested by centrifugation at 4 °C. Cells were washed three times and resuspended in phosphate buffered saline (PBS) buffer.

After cell disruption, the membranes were pelleted by ultracentrifugation at 300,000 *×g* for 1 h. Membranes were solubilized by agitation in PBS buffer with 1% (w/v) DPC and 10 mM imidazole for 1 h. Insoluble material was removed by ultracentrifugation at 200,000 *×g* for 1 h, and the resulting supernatants were loaded onto Ni-NTA agarose (Qiagen). The protein-bound resin was washed 2–3 times using PBS buffer with 30 mM imidazole, 10% glycerol (v/v), and 0.1% LDAO, and eluted with the same detergent buffer with 300 mM imidazole. Purified samples were subjected to size-exclusion chromatography on a Superdex 200 column (GE Healthcare) to purify and assess the homogeneity and stability in 100 mM NaCl, 10% glycerol (v/v), 0.1% LDAO (5×CMC), 10 mM N-(2-hydroxyethyl) piperazine-N’-propane-sulfonic acid (HEPES), pH 7.4.

### Preparation of lipid vesicles

Unilamellar vesicles were prepared by sonicating as described previously [32], [33]. All experiments was used DOPC: DPPC: egg PA: cholesterol at molar ratios of 4:4:1:1. For preparation of thin lipid films, dry lipid mixtures were dissolved in chloroform and phospholipid/chloroform mixtures were dried under a gentle stream of nitrogen. Dried lipid films were placed under high vacuum for 5 h, resuspended in 10 mM HEPES buffer (pH 7.4), vortexed vigorously, and incubated for 30 min at room temperature to completely resuspend. The suspension was sonicated with a non-probe sonicator for 15 min at 300 watt, on 1s and off 0.5 s, in a room temperature water bath.

### Solubilization of preformed liposomes

Liposomes prepared by sonicating were distributed into 1 ml aliquots and solubilized by adding detergent. Solubilization process of liposome is represented by a three-stage model previously described for all types of detergents [30]. DDM, SC, CHAPS and LDAO were tested at various concentrations for their suitability for liposome solubilization. The degree of liposome vesicles dissolution was determined by measuring turbidity with a Ultrospec4300 Pro ultraviolet and visible spectrophotometer (GE, USA) at 400 nm.

### Proteoliposome preparation based on detergent-mediated reconstitution

With increasing detergent, liposome vesicles transitioned from saturation to micelle conditions. After reaching a stable turbidity, monodisperse detergent-protein solutions were added at lipid: protein mass ratio of 20:1 to saturated, partly dissolved, or completely solubilized liposome-detergent solutions in a total volume of 1 ml. Protein-lipid-detergent mixtures were incubated for 50 min at room temperature. Detergent was removed by adsorption on preconditioned Bio-Beads SM-2 [30]. After the removal of Bio-Beads SM-2, proteoliposome solutions were centrifuged at 300,000 ×*g* for 30 min at 4°C and pellets resuspended in 10 mM HEPES buffer. Phospholipid vesicle recovery was performed by phosphorus analysis of the harvested liposomes as described by Bartlett and Bottcher et al [34], [35]. Protein recovery was determined using a standard curve of GFP fluorescence versus the protein concentration and Micro BCA protein assays of the reconstituted protein.

### Determination of the morphology and size of liposome

The average particle size of liposomes and proteoliposomes was determined by dynamic light scattering (DLS, SZ-100, Horiba, Japan). All the lipid solutions were centrifuged at 5000 ×*g* for 10 min before DLS experiments to remove debris. Liposome and proteoliposome morphology was determined by TEM and FM.

For TEM, liposome vesicles were stained in 2% sodium phosphotungstate. Equal quantities of sample and stain (10 µl) were mixed, and a drop of the mixture was placed onto a formvar grid held by tweezers. After 20 s, solution was removed with filter paper and the grid was air-dried. Liposome vesicles were observed under a JEM-2100 TEM (JEOL, Japan) at 100 kV with direct magnification of 40,000×.

For FM, a drop of proteoliposomes was put on a glass slide, covered with a coverslip, and immediately imaged with a fluorescent microscope (Olympus B×51, Japan). Images were processed with DPcontroller ver. 2.2.1.227 software.

### Orientation of AgrC_TM5-6C_ in proteoliposomes

Protein transmembrane topology in proteoliposomes was determined using membrane-impermeable and membrane-permeable thiol-reactive reagents as previously described [23], [36]. After incubation with thiol-reactive reagents, proteoliposomes were washed with 10 mM HEPES (pH 7.4) and centrifuged at 200,000×g for 1 h at 4 °C. All reactions were stopped by addition of 5×Laemmli loading buffer and subjected to SDS-PAGE. Fluorescence of proteins labelled with 5-IAF was visualized with the UVP GelDoc-It Imaging System (Gene, UK) using excitation wavelength of 490 nm and emission wavelength of 510 nm. After fluorescence detection, the same gel was stained using Coomassie Brilliant Blue.

### Proteoliposome stability

Vesicle stability was assessed by determining the particle size of proteoliposomes using DLS and observing the structure of proteoliposomes using FM at indicated intervals. The DLS method was sensitive to vesicle rupture, fusion or aggregation, indicated by vesicle diameter and particle dispersion index (PDI) changes. Extensive vesicle fusion caused an increase in the size distribution and PDI value of the resulting polydisperse vesicle mixture. DLS provided qualitative information about particle size and extent of vesicle fusion. For imaging, AgrC_TM5-6C_-GFP inserted into liposomes was used for fluorescence imaging.

### *In vitro* autophosphorylation assays

Kinase activity of AgrC_TM5-6C_ in LDAO micelles or proteoliposomes was measured using Kinase-Glo Luminescent Kinase Assay Kit. Assay were performed in 96-well white plates in 50 µL kinase reaction volumes containing 20 µg (0.5 pmol) AgrC_TM5-6C_ and 2 µM ATP in 20 mM HEPES (pH 7.4), 10 mM MgCl_2_, 0.1% BSA, 0.1% LDAO, 1 mM DTT. Kinase reactions were incubated for 20 min at 37 °C. For AgrC_TM5-6C_ in LDAO micelles and in proteoliposomes, effects of different concentrations of the signal molecule autoinducing peptide (AIP) were tested with 0.5 pmol protein per 50 µL reaction mixture. AIP and AgrC_TM5-6C_ were incubated at 37 °C for 20 min. Negative controls (blanks) contained no kinase. Following incubation, 50 µL of ATP detection reagent was added to the assay plates, which were incubated at 37 °C for 15 min. Relative light unit (RLU) signal was measured using the Synergy2 Multi-Mode Microplate Reader (BioTek, USA). To quantitatively determine the fraction of functional activity protein, a standard curve of luminescence signal *versus* ATP concentration was prepared.

## Results

### Protein expression and purification

*S. aureus* truncated AgrC_TM5-6C_, a hydrophobic polypeptide of 297 amino acids, has two transmembrane helices connected by a small polar loop that is exposed to the periplasm (Fig. 1B). The AgrC_TM5-6C_ and AgrC_TM5-6C_-GFP extracted from the membrane pellets with 1% (w/v) DPC was purified by Ni-NTA agarose column chromatography and size-exclusion chromatography. Fractions were analyzed by SDS-PAGE (Fig. 1 C) and western blotting (Fig. 1 D). Fig. 1 C showed that protein purity was at least 95%. AgrC_TM5-6C_-GFP showed an aberrant running behavior on SDS-PAGE and western blotting typically observed for GFP structure preservation (Fig. 1 C, Lane 1; Fig. 1 D, Lane 1) [37]. The predicted molecular mass of AgrC_TM5-6C_ was 35.5 kDa (Fig. 1 C, Lane 2; Fig. 1 D, Lane 2).

### Comparison of detergents for liposome solubilization and protein reconstitution

**Figure 2:**
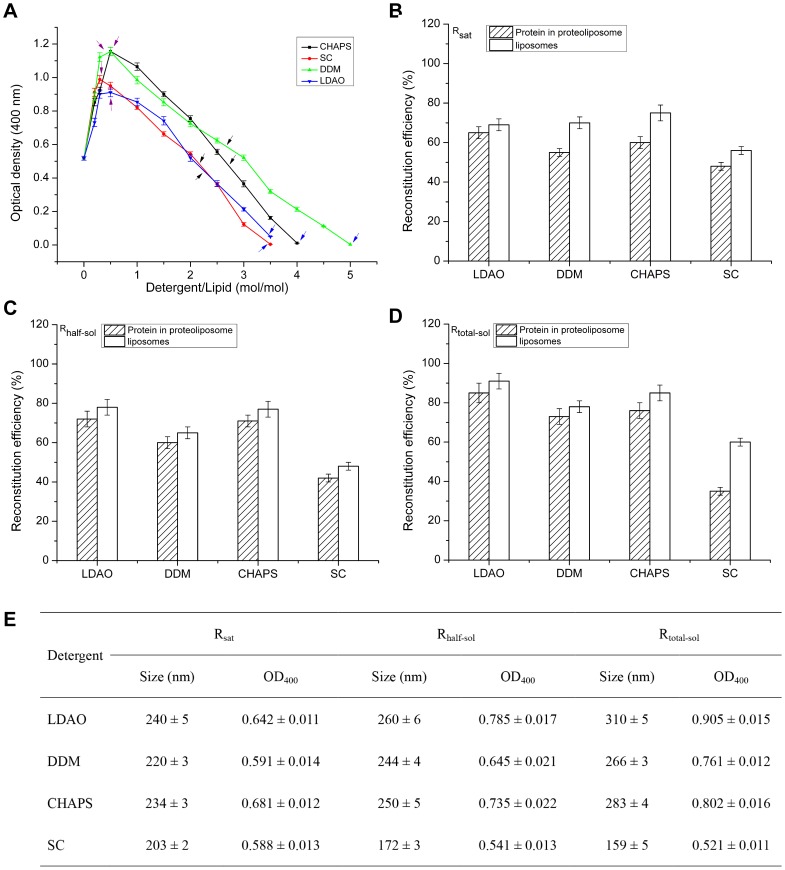
Influence of different detergents on AgrC_TM5-6C_ reconstitution in proteoliposomes. (A) Turbidity of liposome suspensions upon detergent addition. Liposomes prepared by sonicating were resuspended at a final concentration of 2.5 mM in a spectrophotometer cell. Detergents from concentrated stock solutions were added stepwise to liposome suspension under constant stirring at room temperature. Turbidity was measured at 400 nm after equilibration. Detergent threshold concentrations indicated by purple arrows for saturated, black for partially solubilized and blue for completely solubilized liposomes. Results are mean ±S.D. from experiments conducted in triplicate. Effect of detergents on the recovery of lipids and incorporated AgrC_TM5-6C_ at R_sat_ (B), at R_half-sol_ (C), and at R_half-sol_ (D). Size distribution and OD_400_ of AgrC_TM5-6C_ proteoliposomes at R_sat_, R_half-sol_, and at R_half-sol_ (E). R_sat_, detergent-to-lipid ratio in detergent-saturated liposomes; R_half-sol_, detergent-to-lipid ratio in detergent-saturated liposomes and lipid-detergent micelles mixtures; R_total-sol_, detergent-to-lipid ratio in mixed micelles at total solubilization. Data are means of three independent experiments; error bars are SD, P<0.05.

Several commonly available detergents: SC, DDM, CHAPS, and LDAO, were compared for liposome solubilization and protein reconstitution. Incorporation of detergents into bilayers induced turbidity changes (Fig. 2A), depending upon the detergent. In stage I, detergent monomers were incorporated into phospholipid bilayers, saturating the liposomes and resulting in increased turbidity of detergent-liposome mixtures (purple arrows in Fig. 2A). During stage II, structural transitions from detergent-saturated liposomes to small lipid-detergent micelles, resulted in turbidity reduction (black arrows in Fig. 2A). In stage III, all liposomes were completely solubilized and solutions became optically transparent (blue arrows in Fig. 2A). Figure. 2A showed the ability of four detergents to solubilize liposomes. To gain insights into the liposome solubilization process, particle size and morphology of liposomes with detergent LDAO at each stage were measured as a function of the detergent concentration with DLS and TEM (Fig. S1).

To determine the most suitable detergent for AgrC_TM5-6C_ reconstitution, AgrC_TM5-6C_-GFP was added at each step of the liposome solubilization process. Proteoliposomes were reconstituted by removal of detergent from lipid-protein-detergent mixtures. Total phospholipid was determined by phosphorus analysis and the integration of AgrC_TM5-6C_-GFP into the liposomes was measured by GFP standard curve (Fig. S2). Figures. 2B, 2C, and 2D indicate that all detergents tested were capable of reconstituting a certain amount of membrane protein. Following centrifugation, LDAO, CHAPS, and DDM had high efficiency for formation of proteoliposomes in stage III (Fig. 2D). However, recovery of reconstituted protein in proteoliposomes following adsorption of SC was found to be the most effective in stage I, resulting in 48±3% protein recovery and 56±3% liposome recovery (Fig. 2B). Compared with other three detergents, SC gave poor protein reconstruction, possibly because of its ionic nature. Based on reconstitution after complete solubilization, LDAO resulted in 85±5% protein incorporation and 92±4% lipid recovery while stage II yielded about 72±4% protein recovery and 78±4% lipid recovery (Fig. 2C), with stage I leading to recovery of 65±3% protein and 69±4% lipid (Fig. 2B). The turbidity of each proteoliposome stage was recorded as optical density at 400 nm and size distributions of the proteoliposomes were measured by DLS. Figure. 2E shows that larger proteoliposomes particle sizes gave higher OD_400_ values. Turbidity of proteoliposomes was 0.521±0.011 to 0.905±0.015 while the OD_400_ of empty vesicles was 0.506±0.012. The corresponding particle size range of proteoliposomes was from 159±5 nm to 310±5 nm while empty liposomes were about 140±4 nm. Turbidity value might reflect vesicle size. Based on the above analysis, LDAO was used for subsequent experiments.

### Proteoliposome characterization

**Figure 3:**
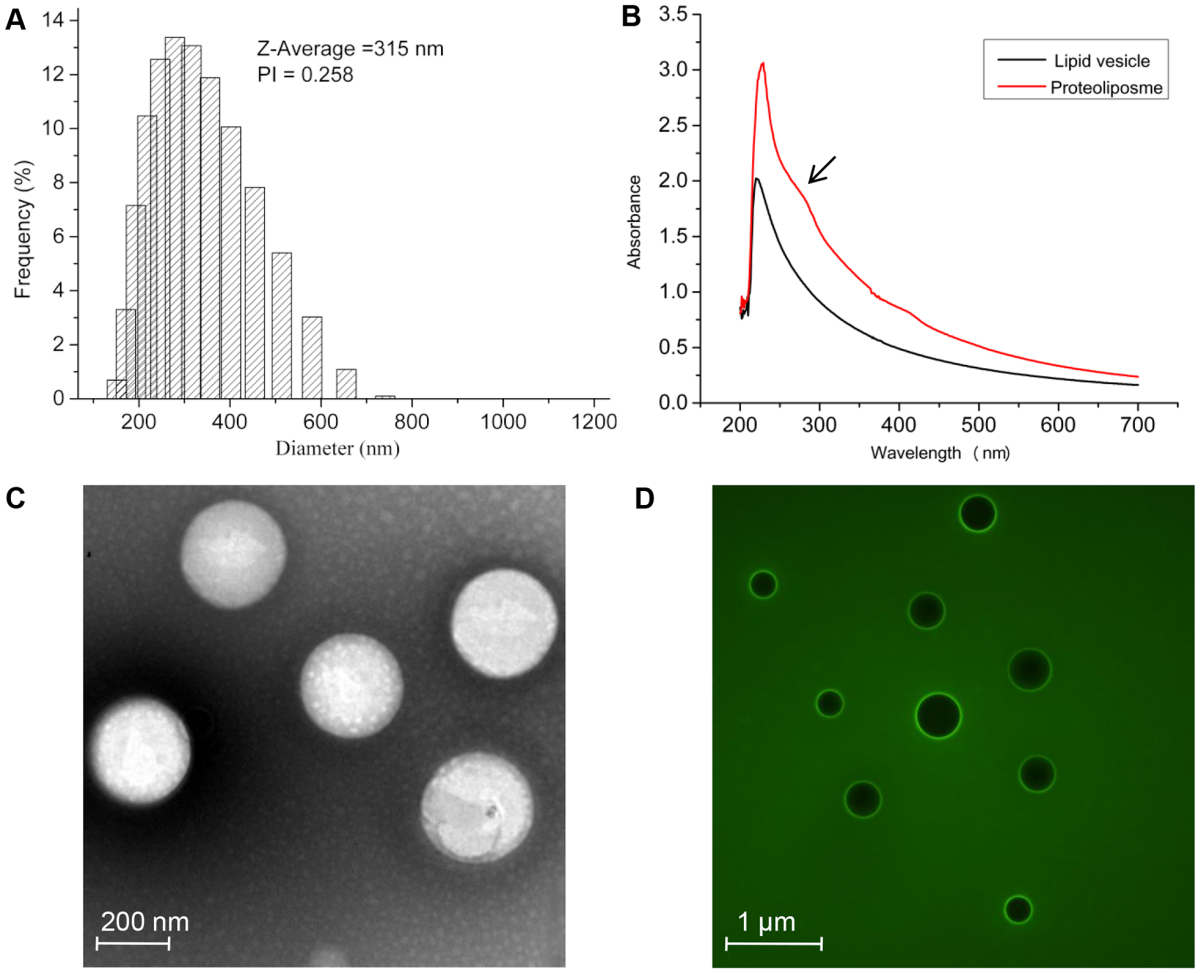
Determination of reconstituted proteoliposomes. (A) Size distribution histogram of reconstituted proteoliposomes. (B) Optical densities of liposomes and proteoliposomes. (C) Electron micrograph of proteoliposomes (D) Fluorescence microscopy dark-field image of proteoliposomes. Fluorescence image of membrane proteins incorporated into liposomes.

To determine if protein was incorporated into the liposome vesicles, we used DLS, which showed proteoliposomes with an average diameter of 315 nm (Fig. 3A), compared to 140 nm for the mean diameter of the empty liposome vesicles was. This result suggested protein reconstitution into a lipid bilayer, which increased vesicle volume and proteoliposome particle diameter. The turbidity of liposome and proteoliposome samples prepared by a detergent-mediated method was recorded between 400 and 700 nm (Fig. 3B). As shown in Figure. 3B, characteristic absorption of proteins in liposomes was 280 nm. This also suggested reconstitution of protein into liposomes. TEM of negatively stained proteoliposomes confirmed structure and morphology. Figure 3C shows the proteoliposome morphology was vesicle of uniform size. Vesicle size measured by TEM was consistent with DLS. To validate TEM observations, we incorporated AgrC_TM5-6C_-GFP into liposomes for observation by fluorescence microscopy. Figure 3D shows proteins in proteoliposome, consistent with the TEM results.

**Figure 4:**
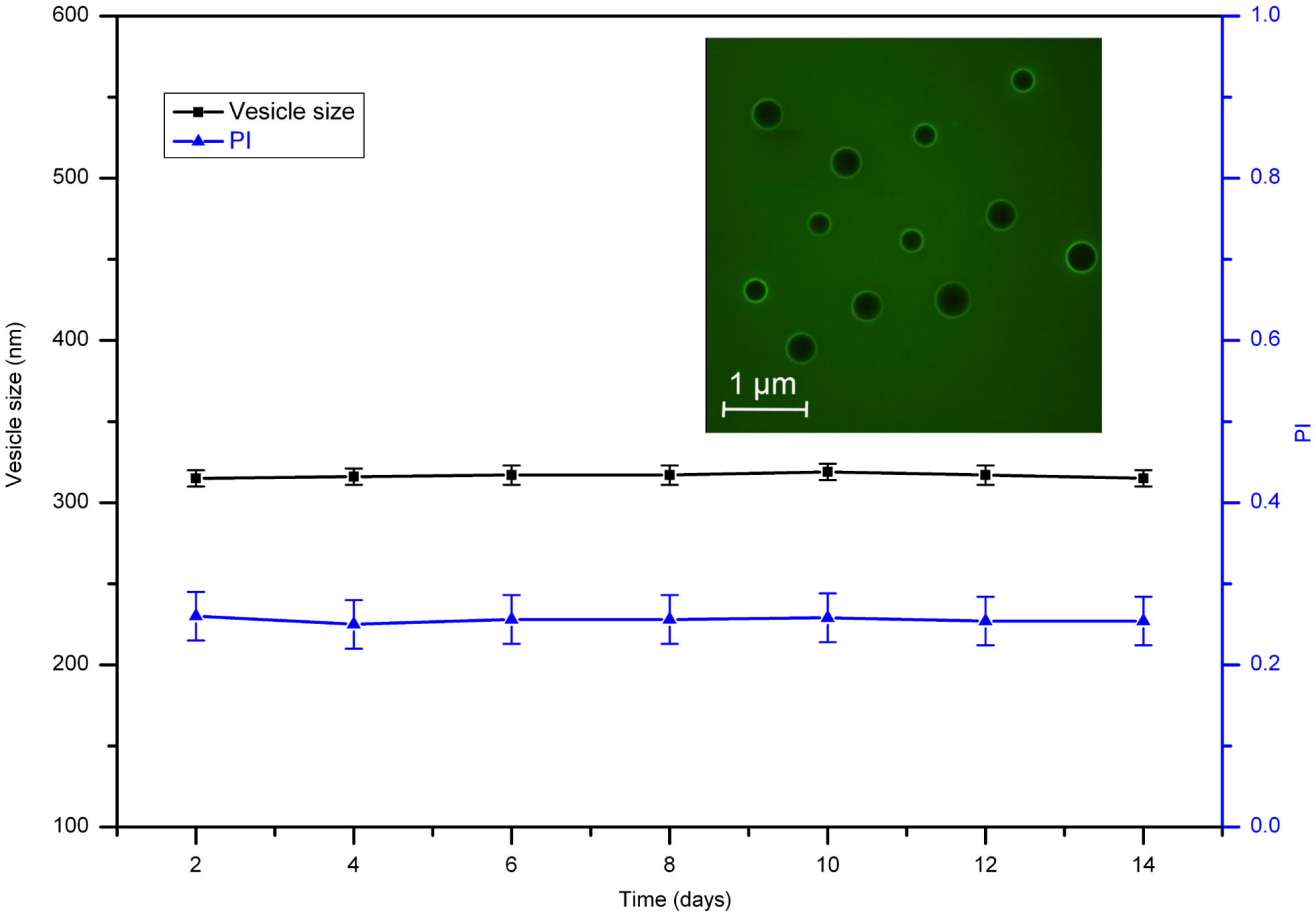
Time course of AgrC_TM5-6C_-GFP proteoliposome stability based on size distribution and PI. Size distribution and PI of proteoliposomes without changes at 14 days after preparation. Inset, fluorescence image with no fusion in samples up to two weeks after preparation. Values represent the mean ± SD of three replicates of three independent experiments.

Proteoliposome stability is associated with storage lifespan. To assess stability, the size distribution and PDI of proteoliposomes was measured on alternate days. Liposomes were stable for at least 14 days (Fig. 4) with no changes in stability after proteoliposome preparation.

### Transmembrane topology of AgrC_TM5-6C_ in proteoliposomes

**Figure 5:**
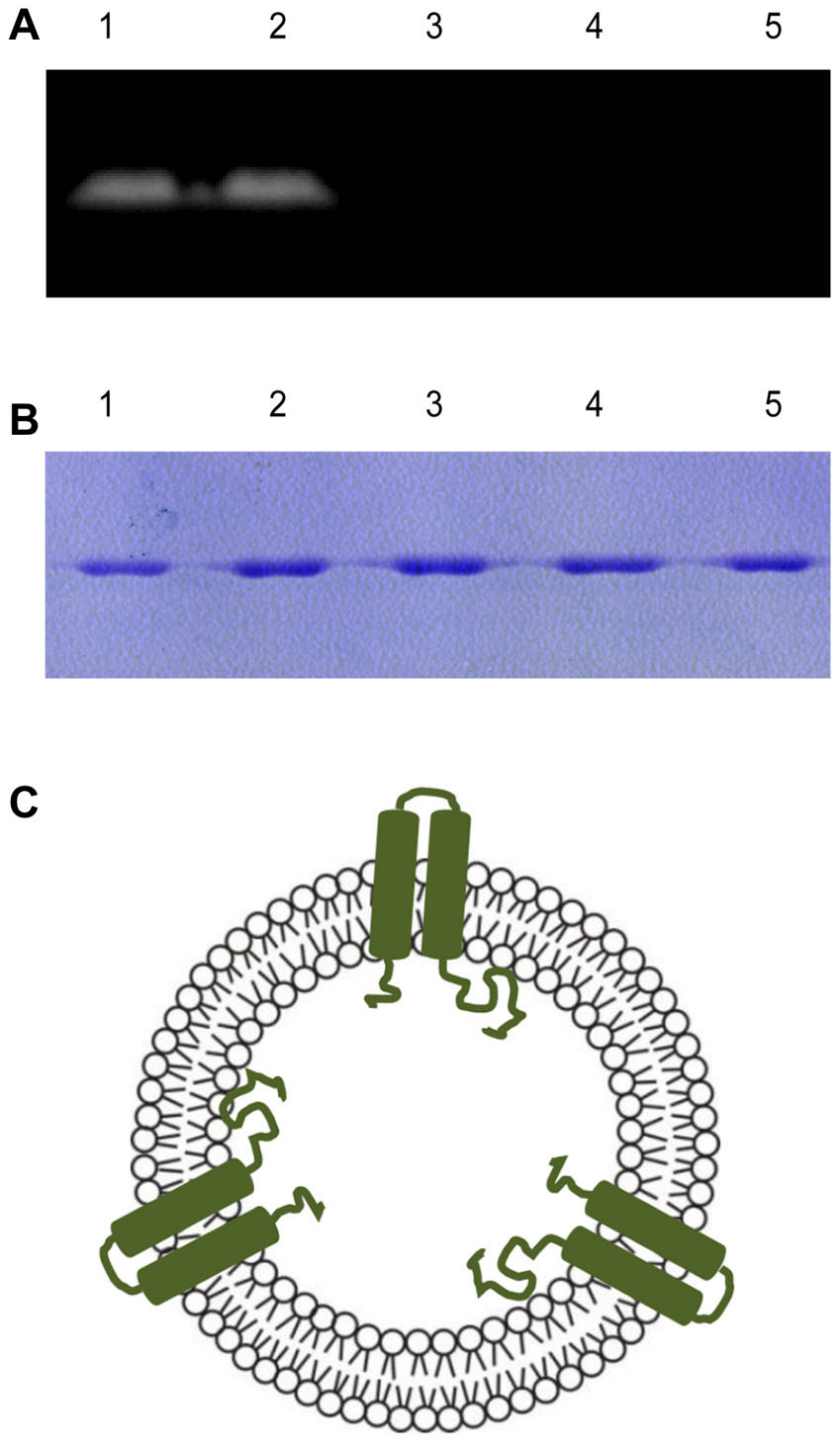
Orientation of AgrC_TM5-6C_ in proteoliposomes. (A) Fluorescence of 5-IAF-labelled AgrC_TM5-6C_. Lane 1, total protein labelling: solubilization of proteoliposomes with LDAO and incubation with 0.33 mM 5-IAF. Lane 2, Internally oriented protein labelling: externally oriented AgrC_TM5-6C_ blocked with 0.33 mM AmdiS followed by solubilization with LDAO and incubation with 5-IAF. Lane 3, Externally oriented protein labelling: proteoliposomes were incubated with 0.33 mM 5-IAF. Lane 4, Nonspecific labelling: proteoliposomes were incubated with 15 mM NEM, then 5-IAF. Lane 5, Solubilized proteins were incubated with 0.33 mM AmdiS, then 5-IAF. All reactions were stopped with 5 × Laemmli loading buffer. (B) SDS/PAGE of total (Coomassie-stained) AgrC_TM5-6C_ in proteoliposomes. (C) Schematic representation of AgrC_TM5-6C_ orientation in proteoliposomes. Experiments were repeated at least10 times under the same conditions.

Next, we determined whether AgrC_TM5-6C_ is inserted into proteoliposomes with a unidirectional transmembrane topology. AgrC_TM5-6C_ has both the NH_2_- and the COOH-terminus in the cytoplasm and a small loop connecting the two hydrophobic helices in the periplasm (Fig. 1B). AgrC_TM5-6C_ contains a unique cysteine at position 337 in its cytoplasmic domain. Total labeling was achieved by solubilizing proteoliposomes with LDAO and labeling the cysteine residue with the membrane-impermeable fluorescent reagent 5-IAF (Fig. 5A, lane 1). Internally oriented protein labeling was determined by blocking the externally oriented AgrC_TM5-6C_ with the membrane-impermeable non-fluorescent reagent AmdiS, solubilizing proteoliposomes with LDAO, and incubating with 5-IAF to label exposed cysteine residues of residual internally oriented AgrC_TM5-6C_ (Fig. 5A, lane 2). The results indicated that more than 95±2% of protein reconstituted into liposomes was internally oriented. To confirm this result, the externally oriented protein labeling was achieved by directly incubating proteoliposomes with the impermeable probe 5-IAF (Fig. 5A, lane 3). Little fluorescence was detected, suggesting that little AgrC_TM5-6C_ was externally oriented in proteoliposomes. In a control reaction for non-specific labeling, proteoliposomes were incubated with the membrane-permeable non-fluorescent thiol-reaction probe NEM followed by 5-IAF (Fig. 5A, lane 4). Little non-specific fluorescence attributable to 5-IAF was found, suggesting specific labeling of the thiol group. Another control reaction was performed by incubating solubilized AgrC_TM5-6C_ with the membrane-impermeable non-fluorescent reagent AmdiS and labelling the cysteine residue with 5-IAF (Fig. 5A, lane 5). No fluorescence was seen, indicating that AmdiS blocked the Cys residues. These results indicated that periplasmic loop of AgrC_TM5-6C_ was localized on the outside of vesicles, while the COOH-terminus was located in the lumen of proteoliposomes. These data showed that AgrC_TM5-6C_ in proteoliposome had acquired a unidirectional membrane topology. Following the fluorescence experiments, SDS-PAGE gels were stained with Coomassie Brilliant Blue to ensure that similar amounts of protein were used in all experiments (Fig. 5B). Together, these results provided evidence that AgrC_TM5-6C_ in proteoliposomes might be able to sense the extraluminal environment through the sensory domain and signal to the intraluminal histidine kinase domain (Fig. 5C).

### *In vitro* autokinase activity of AgrC_TM5-6C_ in proteoliposome

**Figure 6:**
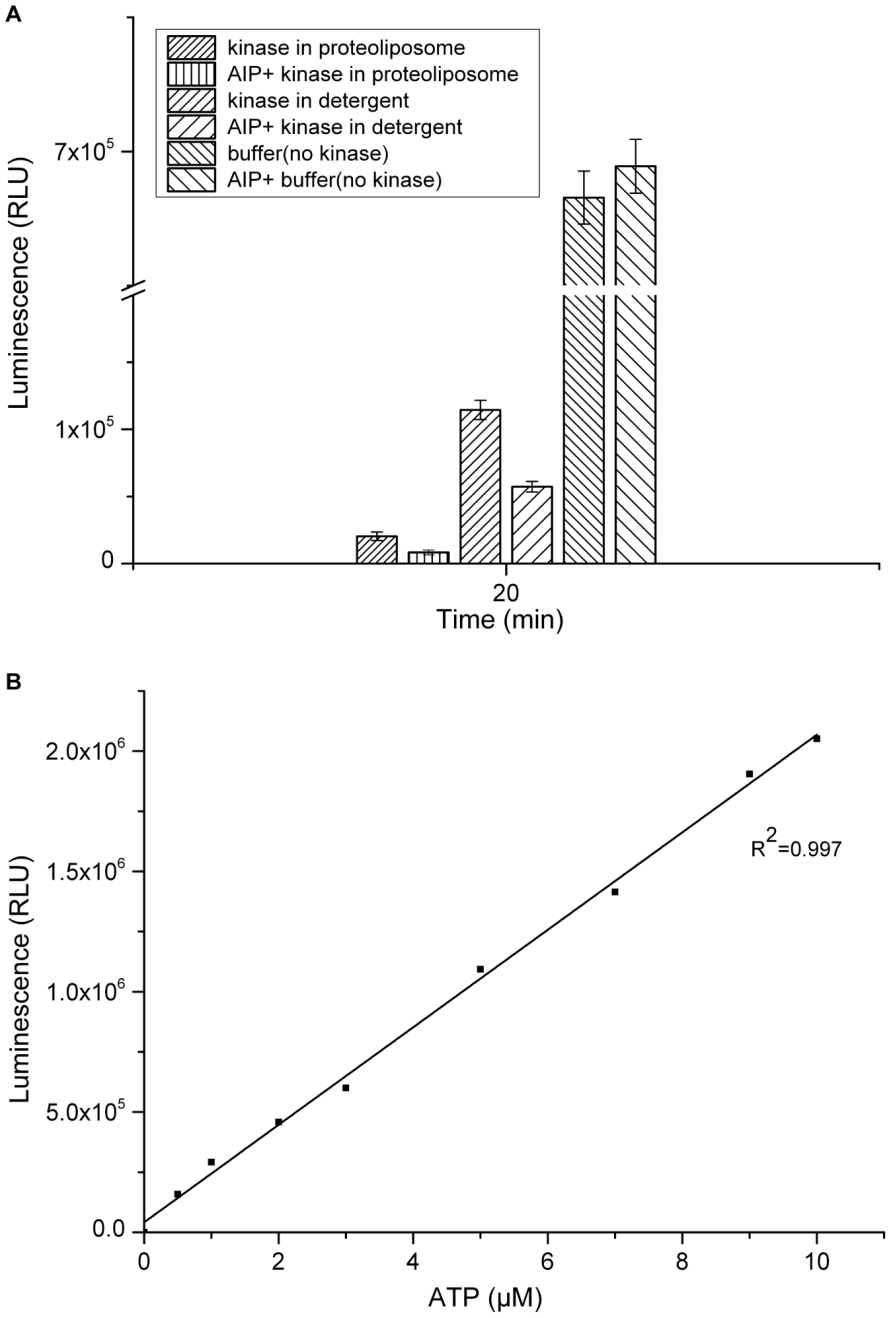
Luminescent kinase assay. AgrC_TM5-6C_ in proteoliposomes and LDAO micelles were resuspended in 50 µl kinase buffer with 2 µM ATP. Reactions with 20 ng (0.5 pmol) AgrC_TM5-6C_ in proteoliposomes and in LDAO micelles were performed with or without 1 pmol AIP. Control, reaction without AgrC_TM5-6C_. Values are mean ± SD of three replicates of three independent experiment. RLU  =  relative light units; P<0.001.

To assess the autokinase activity of reconstituted AgrC_TM5-6C_, the amount of ATP remaining in solution after kinase reactions was quantified. Kinase reactions were carried out for 20 min, followed by addition of an equal volume of Kinase-Glo kit reagent and luminescence measurement (Fig. 6A). AgrC_TM5-6C_ in proteoliposomes and in detergent micelles retained constitutive autophosphorylation activity in the absence of signal molecules. AgrC_TM5-6C_ in proteoliposomes incubated for 20 min showed a 30-fold luminescence reduction compared to samples without kinase (P<0.001), while a 60% reduction was detected for AgrC_TM5-6C_ in LDAO micelles when compared to samples without kinase (P<0.001). A five- to six-fold reduction in luminescence was detected for AgrC_TM5-6C_ in proteoliposomes compared to AgrC_TM5-6C_ in LDAO micelles (P<0.001). These results demonstrated that reconstituted AgrC_TM5-6C_ had autokinase activity.

To determined if the signal molecule AIP induced activation through direct interaction with the AgrC_TM5-6C_, purified AgrC_TM5-6C_ and AgrC_TM5-6C_ proteoliposomes were incubated with different concentrations of AIP for 20 min. Compared with samples without signal molecule, AIP stimulated AgrC_TM5-6C_ autophosphorylation by approximately 2-fold in response to a 2-fold molar excess of AIP over AgrC_TM5-6C_ (Fig. 6A). Higher or lower AIP concentrations elicited no increase in phosphorylated AgrC_TM5-6C_ (data not shown). To determine whether AIP was specific for the AgrC_TM5-6C_ or if it stimulated the activity of other kinase, the effects of AIP on *S. aureus* KdpD kinase were tested. Using different concentrations of AIP did not change activity of KdpD kinase (data not shown), indicating that AIP exerted a specific effect on AgrC_TM5-6C._ These results suggested direct and specific interactions between AIP and AgrC_TM5-6C_. Based on a standard curve of luminescence signal *versus* ATP concentration (Fig. 6B), the fraction of constitutive, functional, active AgrC_TM5-6C_ in proteoliposomes was estimated to be as high as 85% and 65% in detergent micelles in the absence of AIP.

## Discussion

AgrC, a member of the HPK_10_ family, is a membrane protein that is important in signal transduction. Although AgrC has been extensively studied [6], [17]–[21], [38]–[41], biochemical and biophysical studies of AgrC *in vitro* are challenging because of its amphipathic nature and the difficulty of expressing native protein in large amounts. Therefore, in this present study, a truncated AgrC_TM5-6C_ containing the last two transmembrane segments and cytoplasmic domain was constructed and overproduced and purified from an *E. coli* system (Fig.1). AgrC_TM5-6C_-GFP was used for screening detergents to facilitate detection of protein reconstruction efficiency. Although the GFP fusion proteins have been used to monitor bacterial membrane protein expression, screen detergents used for solubilization, and analyze the topological structure of transmembrane protein [42]–[45], it is the first report to test the effects of different detergents on the protein reconstruction efficiency using a GFP fusion protein, which allowed convenient and fast measurements by fluorescence intensity before and after protein incorporation.

AgrC_TM5-6C_ in proteoliposomes showed a constitutive kinase activity (Fig.6A), while AgrC_TM5-6C_ in the presence of detergent revealed relatively low activity. One likely reason for this result is that AgrC_TM5-6C_ in detergent micelles lost their dimeric structure, which is important for autophosphorylation between two AgrC monomers [6]. Another possible reason for this result was that the liposome component was indispensable for kinase activity. Compared with detergent micelles, liposomes might mimic a more natural membrane environment, allowing appropriate conformational changes or structural arrangements for AgrC kinase activity. We demonstrated that autophosphorylation increased upon addition of AIP into activity assays (Fig. 6). This result indicated that the sites for AIP interaction with AgrC were not only in the first two extracellular loops.

Reconstitution of membrane proteins into liposomes provides a model membrane system, which could be important for detailed structure and function studies on membrane proteins. The most successful and frequently used methods for proteoliposome preparation use detergents [28], [30], [31], because most membrane proteins are not soluble in aqueous solution. Detergents are needed to disrupt the structure of native membranes in the initial solubilization step and as a means of sheltering the hydrophobic part of the membrane protein from a water-based environment during further purification. The optimal conditions for AgrC_TM5-6C_ incorporation into liposomes were assessed using a detergent-mediated method [46], [47]. After analyzing the effect of different detergents, LDAO was chosen for AgrC_TM5-6C_ and AgrC_TM5-6C_-GFP reconstitution. Detergent screening is a crucial step in protein reconstitution [48]–[50]. Our results (Fig. 2D) indicated that when the LDAO concentration reached complete solubilization (stage III), recovery of protein and liposomes was the highest. For optimal incorporation, especially for newly studied membrane proteins, we suggest reconstitution considers using different types of detergents over the entire solubilization process: detergent-saturated liposomes, half solubilization of liposomes, and complete solubilization of liposomes. This allows rapid screening of the best detergent for incorporation of the membrane protein, because the efficiency of the final proteoliposomes by a detergent-mediated pathway mainly depends on the detergent used.

A variety of methods developed for liposomes are appropriate for determining average size and the size distribution of reconstituted proteoliposomes. DLS is popular for obtaining information about the size and polydispersity of a reconstituted proteoliposomes. Although measuring the size distribution of reconstituted proteoliposomes by DLS is somewhat non-specific, DLS in combination with TEM, gives valuable information on vesicle size over a range of sizes. The insertion of fluorescently tagged proteins allows recognition by FM, which is practical and straightforward for confirming membrane protein reconstitution.

This study used the phospholipid DPPC because of their low cost compared with other phospholipids. DOPC was used because its gel-to-fluid phase transition of -20 °C was below the room temperature. To provide greater stability, cholesterol was also incorporated into phospholipid mixtures. Charged egg PA or egg phosphatidylglycerol is often also added [30] because these negatively charged lipids prevent liposome fusion or aggregation. In addition, the activity of many membrane proteins depends on negatively charged lipids. In this study, proteoliposomes remained stable for at least two weeks (Fig. 4).

The orientation of the incorporated protein was determined with membrane-permeable and membrane-impermeable thiol-reagents. Figure 5 shows that AgrC_TM5-6C_ was unidirectionally oriented with the COOH-terminus oriented towards the inside of the liposome vesicles. The orientation of incorporated proteins might have been influenced by the detergent removal with Bio-beads SM2 because the rate of detergent removal can be crucial for protein orientation. When detergent is removed quickly, the protein incorporation occurs during formation of the vesicle favoring more symmetry in orientation [51]. Slow removal of detergent leads to preferential formation of liposomes and subsequent protein incorporation, which can favor protein asymmetry in orientation [52]. The membrane composition and protein incorporation stages (stage I, II, III) can also cause protein topology bias [53], [30].

In summary, we prepared proteoliposomes that might be helpful for studying the structure and function of AgrC_TM5-6C_ as well as for illustrating signal transduction mechanisms at the molecular level. Many membrane proteins, such as AgrC, are fully active only when correctly oriented and inserted in a lipid bilayer. Therefore, reconstituting membrane proteins into phospholipid vesicles is useful for structural and functional study [54]–[57]. The results in this paper showed that careful choice of detergent, phospholipid mixture, and the stage at which proteins are added into liposomes to prepare proteoliposomes were essential to optimize protein incorporation. This type of analysis could prove instrumental for studies of structure-function relationships of the pharmacologically important protein AgrC and could also be applicable for other integral membrane proteins. Intensive study of reconstituted proteoliposomes could provide insights for discovering new drug targets and therapeutic agents for treatment of disorders that involve AgrC protein.

## Supporting Information

Figure S1 **Size distribution histograms of liposomes treated by LDAO with electron microscopy images.** (A) Liposome vesicles prepared by sonication had a mean diameters of 140 nm; (B) Saturated-liposomes prepared by LDAO had a mean diameters of 340 nm; (C) partially solubilized-liposomes prepared by LDAO had a mean diameters of 270 nm; (D) completely solubilized-liposomes prepared by LDAO had a mean diameters of 80 nm. Liposomes and detergent-liposome mixtures were examined by TEM after negative staining with 2% sodium phosphotungstate. Inset in S1A, liposomes prepared by sonication that were unilamellar vesicles. Inset in S1B, after detergent addition, suspensions of the large unilamellar vesicles rapidly reached saturation equilibrium, increasing turbidity and particle size. Inset in S1C, revealed system of detergent-saturated vesicles and lipid-detergent mixed micelles caused by detergent partitioning into vesicles, diminishing turbidity and particle size slightly. Inset in S1D, liposome vesicles transformed into mixed micelles with size distribution of 80 nm. Scale bars, 200 nm (A) or 0.5 µm (B, C, and D).
(TIF)

Figure S2 **Fluorescence intensities plotted against GFP-6His.** GFP-6His was overexpressed and purified as described in Methods. GFP concentration was determined by BCA assay and GFP fluorescence was measured with a fluorescence spectrophotometer. Standard curve of GFP fluorescence *versus* protein concentration was used to estimate overexpressed or incorporated membrane protein.
(TIF)
