# Supplementary figures and images for: Functional Reconstitution of Staphylococcus aureus Truncated AgrC Histidine Kinase in a Model Membrane System
Source: PLoS One. 2013 Nov 26;8(11):e80400. doi: 10.1371/journal.pone.0080400 (PMC3841183; doi:10.1371/journal.pone.0080400)

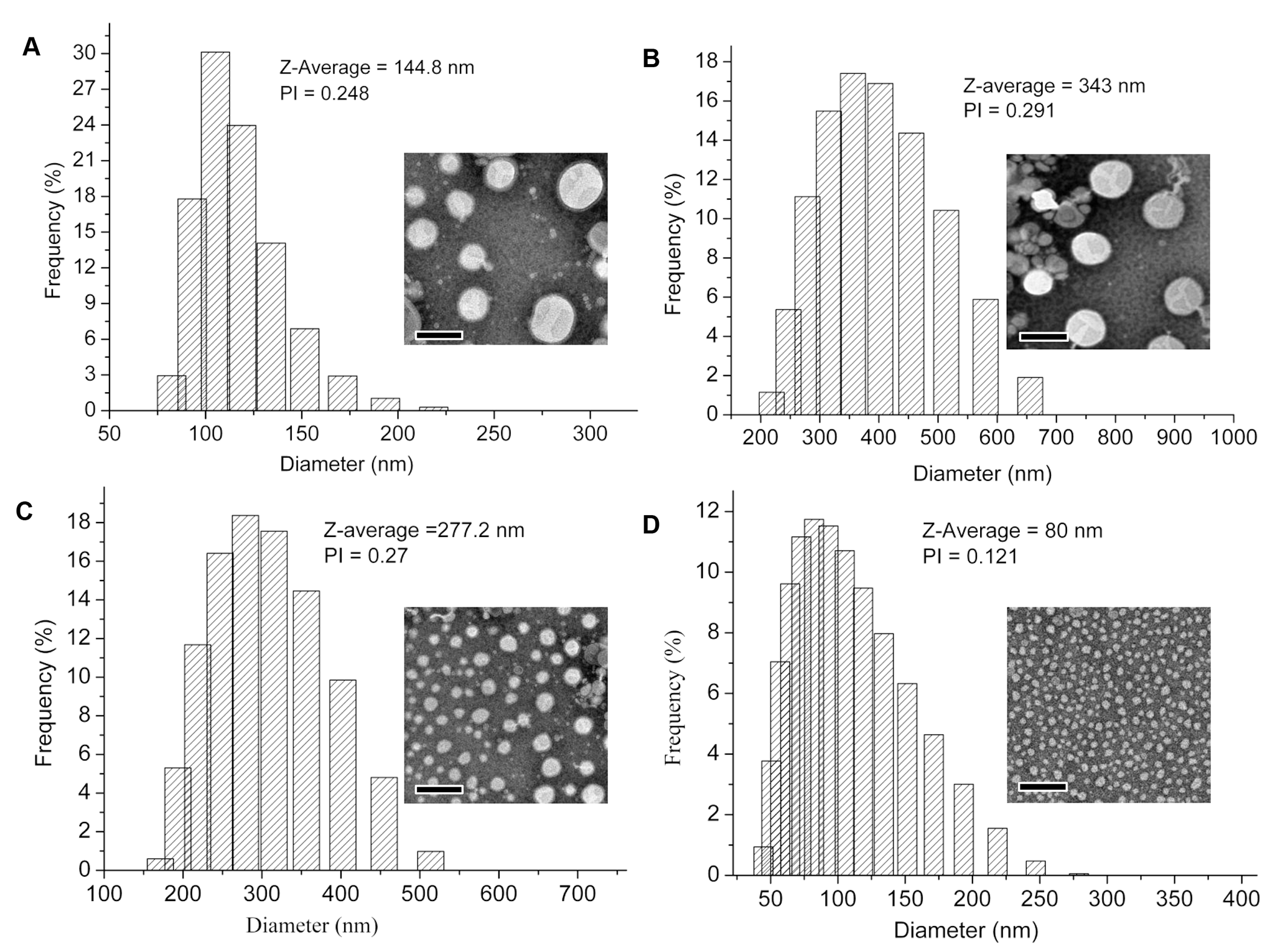

Supplement: Figure S1 — Size distribution histograms of liposomes treated by LDAO with electron microscopy images. (A) Liposome vesicles prepared by sonication had a mean diameters of 140 nm; (B) Saturated-liposomes prepared by LDAO had a mean diameters of 340 nm; (C) partially solubilized-liposomes prepared by LDAO had a mean diameters of 270 nm; (D) completely solubilized-liposomes prepared by LDAO had a mean diameters of 80 nm. Liposomes and detergent-liposome mixtures were examined by TEM after negative staining with 2% sodium phosphotungstate. Inset in S1A, liposomes prepared by sonication that were unilamellar vesicles. Inset in S1B, after detergent addition, suspensions of the large unilamellar vesicles rapidly reached saturation equilibrium, increasing turbidity and particle size. Inset in S1C, revealed system of detergent-saturated vesicles and lipid-detergent mixed micelles caused by detergent partitioning into vesicles, diminishing turbidity and particle size slightly. Inset in S1D, liposome vesicles transformed into mixed micelles with size distribution of 80 nm. Scale bars, 200 nm (A) or 0.5 µm (B, C, and D). (TIF) [file pone.0080400.s001.tif]

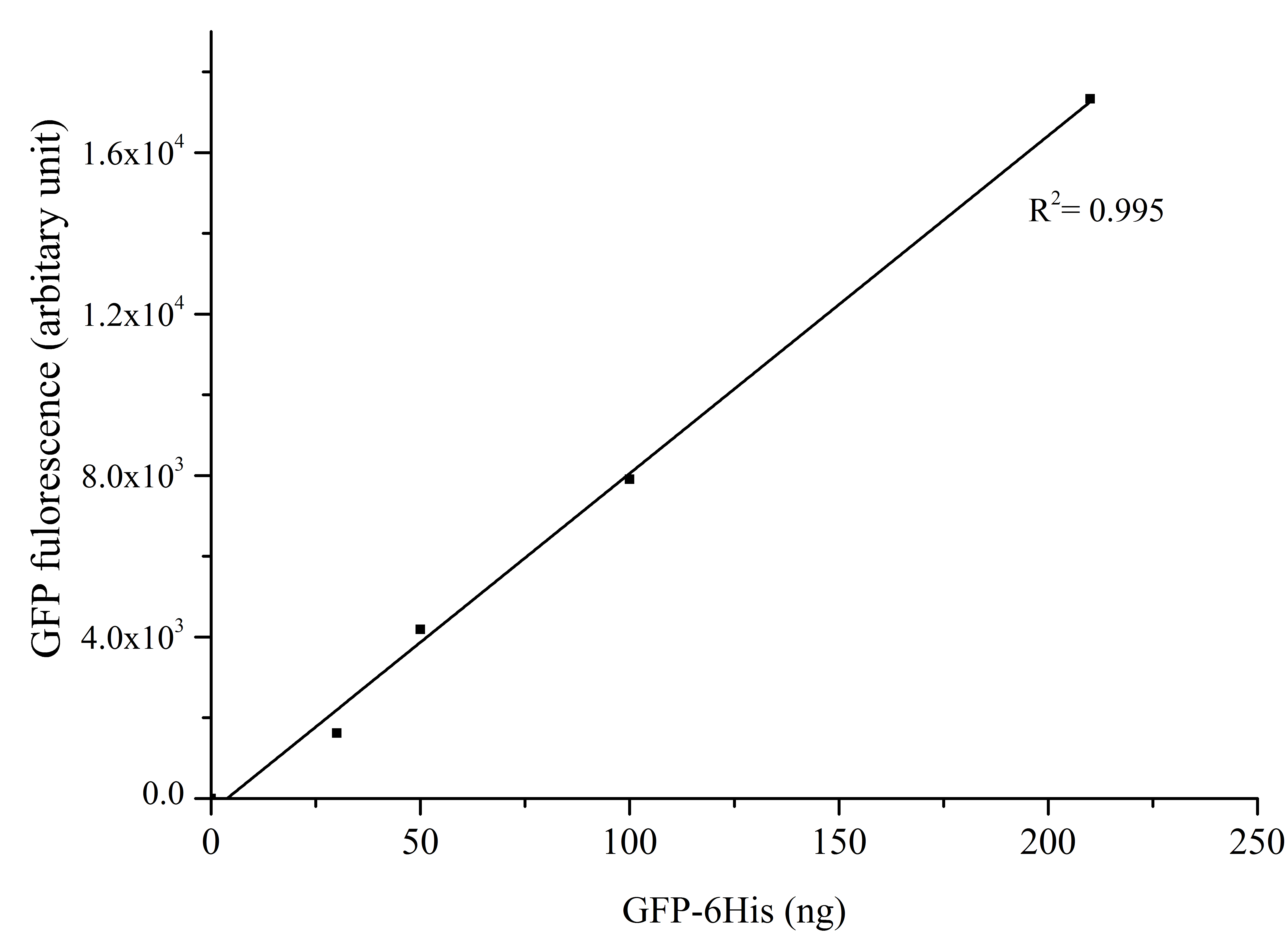

Supplement: Figure S2 — Fluorescence intensities plotted against GFP-6His. GFP-6His was overexpressed and purified as described in Methods. GFP concentration was determined by BCA assay and GFP fluorescence was measured with a fluorescence spectrophotometer. Standard curve of GFP fluorescence versus protein concentration was used to estimate overexpressed or incorporated membrane protein. (TIF) [file pone.0080400.s002.tif]
